# Correlates of Sedentary Behaviour in Adults with Intellectual Disabilities—A Systematic Review

**DOI:** 10.3390/ijerph15102274

**Published:** 2018-10-17

**Authors:** Alyt Oppewal, Thessa I. M. Hilgenkamp, Liselotte Schäfer Elinder, Ellen Freiberger, Pauli Rintala, Myriam Guerra-Balic, Maria Giné-Garriga, Antonio Cuesta-Vargas, Guillermo R. Oviedo, Oriol Sansano-Nadal, Rocio Izquierdo-Gómez, Ingi Einarsson, Antti Teittinen, Craig A. Melville

**Affiliations:** 1 Department of General Practice, Erasmus MC University Medical Center Rotterdam, P.O. Box 2040, 3000 CA Rotterdam, The Netherlands; t.hilgenkamp@erasmusmc.nl; 2 Department of Kinesiology and Nutrition, University of Illinois, 1919 W. Taylor St., Chicago, IL 60612-7256, USA; 3 Department of Public Health Sciences, Karolinska Institutet, Tomtebodavägen 18A, S-171 77 Stockholm, Sweden; Liselotte.Schafer-Elinder@ki.se; 4 Institute for Biomedicine of Ageing, FAU Erlangen-Nürnberg, Kobergerstr. 60, 90408 Nürnberg, Germany; ellen.freiberger@fau.de; 5 Faculty of Sport and Health Sciences, University of Jyväskylä, P.O. Box 35, FI-40014 Jyväskylä, Finland; pauli.rintala@jyu.fi; 6 Faculty of Psychology, Education and Sports Sciences Blanquerna, Ramon Llull University, C. Císter 34, 08022 Barcelona, Spain; miriamelisagb@blanquerna.url.edu (M.G.-B.); mariagg@blanquerna.url.edu (M.G.-G.); guillermorubeno@blanquerna.url.edu (G.R.O.); oriolsn@blanquerna.url.edu (O.S.-N.); 7 School of Health and Life Sciences, Glasgow Caledonian University, Cowcaddens Road, Glasgow G4 0BA, UK; 8 Department of Physiotherapy, University of Málaga, Av/Arquitecto Peñalosa, 3, 29071 Malaga, Spain; acuesta@uma.es; 9 Faculty of Health Sciences Blanquerna, Ramon Llull University, c. Padilla 326-332, 08025 Barcelona, Spain; 10 Department of Physical Education, Faculty of Education Sciences, University of Cádiz, Calle Ancha, 16, 11001 Cádiz, Spain; rocio.izquie@gmail.com; 11 Department of Physical Education, Faculty of Education, Universidad Central de Chile, Edificio Vicente Kovacevic II, Avda. Santa Isabel 1278, Santiago de Chile 8320000, Chile; 12 School of Science and Engineering, University of Reykjavik, Menntavegur 1, 101 Reykjavík, Iceland; ingithore@ru.is; 13 Finnish Association on Intellectual and Developmental Disabilities, Viljatie 4 A, 00700 Helsinki, Finland; Antti.Teittinen@kvl.fi; 14 Institute of Health and Wellbeing, University of Glasgow, 1055 Great Western Road, Glasgow G12 0XH, UK; Craig.Melville@glasgow.ac.uk

**Keywords:** sedentary lifestyle, physical inactivity, determinants, health promotion, developmental disabilities

## Abstract

Individuals with intellectual disabilities (ID) are at high risk for high levels of sedentary behaviour. To inform the development of programmes to reduce sedentary behaviour, insight into the correlates is needed. Therefore, the aim of this study is to review the evidence on correlates of sedentary behaviour in adults with ID. We performed a systematic literature search in Ovid Medline, Ovid Embase, Web of Science and Google Scholar up to 19 January 2018, resulting in nine included studies that were published from 2011 to 2018. Correlates were categorized according to the ecological model. Studies predominantly focused on individual level correlates. Of those correlates studied in more than one study, having epilepsy was associated with less sedentary behaviour and inconsistent results were found for sex, genetic syndromes, weight status, physical health, mobility, level of ID, and mental health. Of the few interpersonal and environmental factors studied, only living arrangements were studied in more than one study, with inconsistent results. To date, we have limited and inconclusive evidence about correlates of sedentary behaviour in adults with ID. Only when future studies unravel correlates and determinants, across all domains of the ecological model, will the potential opportunities to improve health by reducing sedentary behaviour come within reach.

## 1. Introduction

Reducing sedentary behaviour is one of the new promising strategies to promote a healthy lifestyle and improve health [1,2,3]. The *Sedentary Behaviour Research Network* defines sedentary behaviour as any waking behaviour with an energy expenditure ≤ 1.5 metabolic equivalents (MET), while in a sitting, reclining or lying posture [4]. Prolonged sitting results in cardiovascular and other health risks, independent of the risk associated with a lack of physical activity or exercise [5,6,7].

Individuals with intellectual disabilities (ID) are a population particularly at risk for the health consequences of high levels of sedentary behaviour. An intellectual disability is defined by a significant limitation in both intellectual functioning and adaptive behavior, originating before the age of 18 [8]. In a meta-analysis of population-based studies, the prevalence of ID was estimated to be 1% of the total population, with the highest prevalence in low- and middle income countries [9]. Even though this is a small portion of the population, the health care costs for this group are very high. A study in the Netherlands showed that the health care costs for individuals with ID represent 9% of the total Dutch health care costs [10]. In the USA, the lifetime costs of ID were estimated to be 51.2 billion dollars [11]. Health promotion interventions are therefore of utmost importance for this population.

A recent review showed that adults with ID are at increased risk for high levels of sedentary behaviour than the general population [12]. It also stated that the prevalence was probably even underestimated because of the use of measurement methods not validated in this population, or methods not adapted to the specific living circumstances of individuals with ID [12]. Combining this with previous findings demonstrating higher prevalences of cardiovascular disease, multimorbidity and frailty in individuals with ID [13,14], sedentary behaviour is a potentially valuable target for health promotion interventions in individuals with ID.

Sedentary behaviour is not the same as insufficient physical activity behaviour, which is termed physical inactivity. Compared to physical inactivity, the efforts to reduce sedentary behaviour may take place in different settings, and a different set of correlates may be important to address [1,15]. In the general population, research has also shown that these correlates may be specific for the population of focus. For example, recent systematic reviews reported differences in the correlates of sedentary behaviour in adults (18–65 years; [16]) and older adults (≥65 years; [17]). Because individuals with ID differ from the general population with regard to several aspects, such as ID-related health conditions, genetic syndromes, and a different experience of their environment, it is possible that adults with ID may well have unique correlates of sedentary behaviour relevant to the development of effective behaviour change interventions.

A review on sedentary behaviour in older adults who did not have ID also identified that included studies focused mostly on individual correlates, such as sex and age [17]. Because sedentary behaviour is not only influenced by factors related to the individual, but also by interpersonal and environmental factors, this warrants further research into these factors as well [1,17,18]. In addition, the authors highlighted the need to study how different factors interact with different subdomains of sedentary behaviour, such as occupation, transport, household and leisure time [17].

Such knowledge is key to informing current and future research on this topic, and to identify the gaps of knowledge required to design effective interventions that are adapted to the specific living and working circumstances of adults with ID. However, for adults with ID, no systematic evaluation of the existing evidence is currently available. The aim of the current paper is therefore to provide a systematic review of the evidence on individual, interpersonal, and environmental correlates of sedentary behaviour in adults with ID.

## 2. Methods

We used the Preferred Reporting Items for Systematic Reviews and Meta-Analyses (PRISMA) statement to structure this review [19] (see Table S1). Our review protocol was registered with the PROSPERO international prospective register of systematic reviews (registration number CRD42015025257), as part of the protocol of our previous review regarding measurement and prevalence of sedentary behaviour in adults with ID [12].

### 2.1. Search Strategy

On 19 January 2018, we searched in the databases Ovid Medline, Ovid Embase, Web of Science and Google Scholar. Our search strategy was developed with the help of a biomedical information specialist and search terms on the following topics were included: (a) intellectual disabilities, (b) sedentary behaviour and synonyms (e.g., sedentary lifestyle), and (c) types of sedentary behaviour (e.g., TV viewing time, screen time, computer games; see Table S2). To find any additional relevant studies, we hand searched reference lists and checked on Google Scholar which papers had cited the final records.

### 2.2. Selection of Studies

Studies with an observational (cross-sectional, case-control and prospective), experimental (randomised controlled and quasi-experimental) and qualitative study design were all eligible for inclusion.

Studies had to meet the following inclusion criteria:

(a) Study sample with participants with ID.
(b) Study sample with participants aged ≥18 years. For studies that also included individuals under 18 years, at least 80% of the total sample had to be ≥18 years.
(c) In study samples with mixed developmental disabilities and data only presented for the sample as a whole, at least 50% of the sample had to have an ID. Studies were excluded if they did not report the proportion of participants with ID.
(d) Sedentary behaviour was measured with objective and/or subjective methods.
(e) Correlates of sedentary behaviour are reported.
(f) Studies are published in English.
(g) Studies are published after 1 January 1990.

Studies were excluded if:

(a) It was a conference abstract.
(b) It was a lab-based study e.g., to calibrate accelerometer cut-offs.
(c) The term sedentary was used to describe a lack of physical activity e.g., <5000 steps per day.

After the database search, studies were screened for inclusion with the inclusion and exclusion criteria. First, the first and last author (AO and CM) independently screened the title and abstracts of all identified records, with 98.6% agreement and a Cohen’s kappa of 0.81. Disagreement about inclusion and exclusion was resolved through a consensus discussion. Second, the first and last author (AO and CM) independently read the full text and completed inclusion and exclusion checklists for each paper. Again, disagreement was resolved through a consensus discussion. There was 99.1% agreement and a Cohen’s kappa of 0.94.

### 2.3. Data Extraction and Synthesis

Data was extracted with an adapted version of the data extraction form we previously developed for our review regarding sedentary behaviour in adults with ID [12]. We extracted data on setting, target population, study design and aim, sample characteristics (sample size, age, sex, level and causes of ID), sedentary behaviour measurement and outcome, and correlates of sedentary behaviours (Table 1). In case of experimental studies, only baseline data were used to study the correlates of sedentary behaviour. The first and last author (AO and CM) independently extracted data from all included studies. Disagreements were solved through consensus discussion.

Data were synthesized based on the ecological model of sedentary behaviour [1], as has been done previously in the general population [17,18]. Data were presented according to individual factors (divided into physical, biological, and genetic factors, behavioural factors, and socioeconomic status), interpersonal factors, and environmental factors (Table 2).

### 2.4. Quality Assessment

The Standard Quality Assessment Criteria for Evaluating Primary Research Papers from a Variety of Fields was used to assess the quality of the included articles [29]. This tool contains a separate checklist for qualitative and quantitative studies. Criteria can be scored as ‘yes’ (2), ‘partial’ (1), ‘no’ (0) and not applicable. We calculated a summary score as the sum of the scores on the applicable criteria divided by the maximum possible score, resulting in a summary score in the range of 0–1.0 (Table 1). A higher score represents better quality. The first and last author (AO and CM) independently assessed the quality of all included articles, and disagreements were resolved through a consensus discussion.

## 3. Results

**Figure 1:**
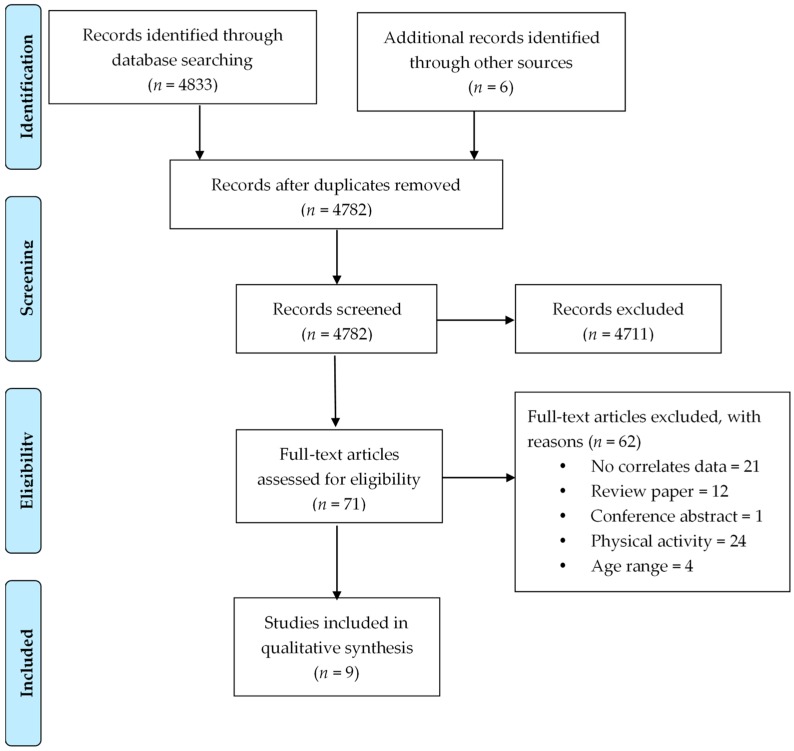
Flow diagram of the study selection process.

The study selection process is shown in Figure 1, with the number of articles retrieved and included at each stage. Most of the full-text articles were excluded because they did not report any correlates of sedentary behaviour or they reported a lack of physical activity instead of sedentary behaviour.

### 3.1. Study Characteristics

Table 1 provides the characteristics of the nine papers included in the data synthesis and the quality ratings for the individual studies.

Eight of the nine included studies used a cross-sectional design, with three studies reporting findings from population-based samples in the UK [27] and the USA [21,26]. The mean age of participants ranged from 28.5–45.0 years.

Four studies used an objective measure of total sedentary behaviour, using an accelerometer [24,25,28] or inclinometer [20]. The other five studies used questionnaires to measure time spent viewing TV/ using computers [21,22,23,26,27], as a proxy definition of sedentary behaviour.

### 3.2. Correlates of Sedentary Behaviour

Table 2 gives an overview of the 25 correlates of sedentary behaviour studied, grouped using the individual, interpersonal and environmental levels in the ecological model [1].

### 3.3. Individual Correlates

All nine studies examined one or more of the 22 individual level correlates of sedentary behaviour, which fitted into physical, biological and genetic (sex, age, genetic syndromes, and all sort of health indicators; nine studies), behavioural (physical activity, Special Olympics participation; two studies) and socioeconomic status (socioeconomic deprivation, employment; three studies) categories of the ecological model [1]. None of the behavioural or socioeconomic factors were correlated with sedentary behaviour.

### 3.4. Physical, Biological and Genetic Factors

None of the 17 factors were examined as a correlate of sedentary behaviour in all nine studies. Age was not correlated with sedentary behaviour in any of the four studies that included it as a variable in the analyses [25,26,27,28].

Three of the five studies that assessed the association between sex and sedentary behaviour reported a statistically significant correlation [20,26,27]. One study found that women [20] were more sedentary, whilst two studies found that men [26,27] were more sedentary. Three studies assessed the association between genetic syndromes and sedentary behaviour [24,26,27]. One study found that individuals with Down syndrome were less sedentary than individuals with Williams syndrome and Prader Willi syndrome [24], whilst the other two studies did not find any association between Down syndrome and sedentary behaviour.

Weight status was the most commonly examined health indicator investigated as a potential correlate of sedentary behaviour. Three studies found that individuals with obesity had higher levels of sedentary behaviour [25,26,27]. However, the study of sedentary behaviour in adults with Down, Williams and Prader Willi syndromes reported that individuals who were underweight or of normal weight were more sedentary than those who were overweight or obese [24]. Three other studies did not find a significant correlation between sedentary behaviour and weight status [21,23,28].

Both studies that used data on screen time/TV viewing from population-based samples of adults with ID found that individuals with epilepsy were less sedentary than individuals without epilepsy [26,27]. These studies both also reported that individuals with more severe ID were less sedentary than individuals with mild ID. However, there were inconsistency for mobility problems; participants with mobility problems were more sedentary in one study [27] whilst the other study did not find any significant correlation [26].

### 3.5. Interpersonal and Environmental Correlates

Of the three correlates (living arrangements, social participation, residential location) assessed from these levels of the ecological model, only living arrangements (residential type, such as own home, family home, group homes, residential care setting) were found to be correlated with sedentary behaviour in one study [26]. However, this finding was not replicated in the three other studies that examined living arrangements as a potential correlate of sedentary behaviour [24,27,28].

## 4. Discussion

This systematic review is the first to investigate the evidence on correlates of sedentary behaviour in adults with ID. Very few studies have investigated factors associated with sedentary behaviour, and therefore to date we have minimal understanding of the correlates of sedentary behaviour in adults with ID. The nine included studies focused predominantly on correlates related to the individual level. Of the individual correlates studied in more than one study, having epilepsy was associated with lower levels of sedentary behaviour. Inconsistent results were found for sex, genetic syndromes, weight status, physical health, mobility, level of ID, and mental health. Of the few interpersonal and environmental factors studied, only living arrangements were studied in more than one study, with inconsistent results.

The focus on research on individual factors is in stark contrast with the importance and with recent emphasis on interpersonal and environmental factors in research in the general population [16]. To be able to reduce sedentary behaviour, an understanding of the multiple correlates operating at different levels is needed. Focussing only on the individual factors will most probably not result in a reduction of sedentary behaviour because the behaviour of an individual is also influenced by its social relationships and its environment [1]. Therefore, to design effective interventions to reduce sedentary behaviour, we need a more thorough insight into the correlates and determinants across all the domains of the ecological model.

None of the studies found a correlation with age in contrast to studies in the general population often showing increasing sedentary behaviour with increasing age [16]. It may be that the high levels of sedentary behaviour of people with ID are established during childhood and maintained into adulthood. This hypothesis is supported by previous studies comparing adolescents with ID with peers without ID, showing that the former are more sedentary [30,31]. In the general population, sedentary and physical activity behaviour during childhood and adolescence seem to track, to some extent, into adulthood [32,33]. Therefore, it seems very important to try to reduce sedentary behaviour already at a young age in individuals with ID, which may then also transfer into less sedentary behaviour in adulthood.

Weight status was most commonly examined as a potential correlate of sedentary behaviour. This may be explained by the high prevalence of obesity in adults with ID, even among Special Olympics athletes, and specifically in females [34,35]. Additionally, successful interventions to reduce weight are scarce and highly needed [34]. Reducing sedentary behaviour may be a successful intervention to reduce obesity, and may be more feasible than, for example, trying to increase moderate to vigorous physical activity within this population. Sparling et al. (2015) recognized that most adults find it difficult to meet physical activity guidelines of ≥150 min of moderate physical activity per week [36]. Whilst recognizing the importance of the physical activity guidelines, a different approach to help adults with ID to become more active could be to encourage reduced sitting and increased light intensity physical activity levels.

However, we found inconsistent results regarding the correlation between weight status and sedentary behaviour across studies. Three studies found obese individuals to be more sedentary [25,26,27], one study that specifically looked at genetic syndromes found individuals who were underweight or of normal weight to be more sedentary [24], and three studies did not find a significant correlation [21,23,28]. In the general population, inconsistent results regarding the association between weight status and sedentary behaviour are also seen [16,17,37]. We therefore need more research to unravel the relationship between weight status and sedentary behaviour, and the potential of reducing sedentary behaviour in weight management programmes.

Unexpectedly, having epilepsy was found to be associated with being less sedentary [26,27]. However, in another study, having epilepsy was identified as one of the predictors for physical inactivity [38]. Even though sedentary behaviour is distinct from physical inactivity, these two results seem contradictory. An explanation may be in the fact that both studies looking at sedentary behaviour and epilepsy used TV viewing/screen time as a proxy measure for sedentary time. Because TV viewing is a common cause of photogenic epilepsy, it could be that individuals with epilepsy are restricted in their screen time [39]. Future studies should take this aspect into account because this could hamper the use of screen time as a proxy measure for sedentary behaviour in this subgroup.

More severe ID was also associated with being less sedentary [26,27], while studies show that individuals with more severe ID seem to be more inactive than individuals with less severe ID [40]. People with more severe ID may be less likely to watch TV because of sensory impairments, difficulties with processing stimuli, and complex impairments in cognition and communication. Screen time may therefore also be less valid as a proxy measure for sedentary behaviour for this subgroup.

Few studies have looked at interpersonal and environmental factors correlated with sedentary behaviour of adults with ID. However, because adults with ID experience their environment differently than the general population, it is especially important to get more knowledge about the influence of interpersonal and environmental factors on sedentary behaviour, specific to this population. The studies included in this review only looked at living arrangements (residential type), social participation (engagement in social activities, Special Olympics participation) and residential location (urban/rural), which is a very limited set of interpersonal and environmental factors. To be able to develop successful interventions to decrease sedentary behaviour in adults with ID more studies are needed on the influence of additional interpersonal and environmental factors on sedentary behaviour, as well as on their interaction.

Additionally, even though the association between individual factors has been studied more widely than the interpersonal and environmental factors, we still have limited knowledge on which individual factors are correlated with sedentary behaviour because we see contradicting results across studies. A better understanding of the influence of individual factors on sedentary behaviour is needed to be able to tailor interventions to individual characteristics. For example, we have limited knowledge on the influence of genetic syndromes on sedentary behaviour, and comorbidities often seen in individuals with ID, such as physical impairments, medication use, and mental health problems.

## 5. Strengths and Limitations

Strengths of this review were closely following the PRISMA guidelines, and performing the process of inclusion of papers and data extraction in duplicate to maximize reliability. The studies included had fairly large sample sizes, and some were population based samples. However, generalizability of the results is limited because of the few studies included and the inconsistency of results.

This review is a continuation on a previous review performed by the same group (the European Network of Physical Activity Research in People with Intellectual Disabilities (ENPARID)) regarding definitions, measurements and prevalence of sedentary behaviour in adults with intellectual disabilities [12]. The current review adds to the previous review by presenting the current knowledge about the correlates of sedentary behaviour, thereby providing a comprehensive view on the current state of research regarding sedentary behaviour in adults with ID.

In a review on correlates of sedentary behaviour in the general population, the associations differed depending on whether sub-domains or total sedentary behaviour time were used [17]. For example, a high amount of sedentary time during transportation may be influenced by different factors than TV viewing time. The studies included in this review looked at total sedentary time and TV or screen time. It is therefore important to keep in mind that these results may not be generalizable for other sub-domains of sedentary behaviour, and that these subdomains may be different for this specific population.

## 6. Future Research

Large-scale epidemiological studies are needed to examine possible correlates and determinants of sedentary behaviour and develop the necessary understanding to develop successful interventions to improve the health of individuals with ID. The focus should be on all the domains of the ecological model, and the complex interplay between individual, interpersonal and environmental factors.

## 7. Conclusions

There is only fragmented information available on correlates of sedentary behaviour in adults with ID, and therefore there is very limited and inconclusive evidence about relationships between correlates and sedentary behaviour. Only when future studies unravel correlates and determinants of sedentary behaviour will the potential opportunities to improve health by reducing sedentary behaviour in this vulnerable group come within reach.

## Figures and Tables

**Table 1:** Characteristics of studies reporting correlates of sedentary behaviours in adults with intellectual disabilities.

Authors	Setting and Target Population	Study Design	Study Aim	Participants					Sedentary Behaviour Measure: Outcome	Potential Correlates Investigated	Quality Score (0–1.0)
				Sample Size	Mean Age (SD ^a^, Range)	Sex (% F ^b^)	Level of ID ^c^	Causes of ID			
Finlayson et al. (2011) [20]	Scotland, adults with mild-moderate ID, living in the community	cross sectional, convenience sample	Collect pilot data on habitual physical activity and inactivity, and compare activity monitor data with self-report data	*n* = 62	37.1 (12.8, 18–66)	56.5%	N/A	9.7% DS ^d^	Total sedentary time by AcvtivPal (sedentary cut-off N/A): 18.71 h/day (SD 1.88, range 14.88–22.19)	Sex	0.82
Hsieh et al. (2014) [21]	USA, adults with all levels of ID, known to specialist organisations	cross sectional, population-based sample	Examine the relationship between nonmodifiable and modifiable risk factors and obesity	*n* = 1619	37.1 (14.1, 18–86)	44.8%	13.3% borderline, 31.6% mild, 23.7% moderate, 8.6% severe/profound, 22.8% unknown	24.9% DS	Hours of TV ^e^ watching (Proxy rater question): Mean sedentary time not reported	Weight status	0.95
Mikulovic et al. (2014) [22]	France, adults with ID, living in institutions	cross sectional, administrative sample	Explore the relationship between sleep habits and overweight/obesity, physical activity and sedentary behaviour	*n* = 691	38.1 (10.3, 19–59)	41%	N/A	N/A	Total hours TV and computer/week (Questionnaire adapted from French Federation Adapted Sports-proxy rater): group 1 20.25 (SD 12.25), group 2 17.75 (SD 12.76), group 3 23.82 (SD 14.89), group 4 27.20 (SD 17.46)	Sleep habits	1.0
Mikulovic et al. (2014) [23]	France, adults with ID, living in institutions	cross sectional, administrative sample	Asess the prevalence of overweight/obesity, and lifestyle, food habits, physical activities and self-awareness about body and health, and assess associations with overweigh/obesity	*n* = 691	N/A	N/A	N/A	N/A	Total hours TV and computer/week (Questionnaire adapted from French Federation Adapted Sports-proxy rater): 18 (12–28)	Weight status	1.0
Nordstrom et al. (2013) [24]	Norway, adults with DS, WS ^f^ and PWS ^g^ living all over Norway	cross sectional, convenience sample	Describe levels of physical activity and sedentary behaviour, and study physical activity and walking capacity in relation to BMI	*n* = 96	28.5 (7.5)	62.1%	N/A	41.7% DS, 29.2% WS, 29.3% PWS	Total sedentary time by ActiGraph GT3X+ accelerometer (sedentary cut-off < 100 cpm): 522 min/day (SD 80.3)	Sex, genetic syndrome (DS, WS and PWS), BMI ^h^, living situation (supported community setting vs with parents)	0.90
Oviedo et al. (2017) [25]	Spain, adults with ID	cross sectional, convenience sample	Assess the temporal patterning of sedentary behaviour and physical activity levels throughout the week, and analyze age and sex differences	*n* = 92	45.0 (12)	41.7%	32.6% mild, 37.0% moderate, 30.4% severe	14.1% DS, 2.2% West syndrome, 2.2% Cerebral Palsy, 2.2% Cornelia Lange syndrome, 1.1% microcephaly	Total sedentary time by ActiGraph GT3X+ accelerometer (sedentary cut-off < 100 cpm): 612.9 min/day (SD 80.1)	Age, sex, center time, BMI	0.64
Hsieh et al. (2017) [26]	USA, adults with all levels of ID, known to specialist organisations	cross sectional, population- based sample	Assess the prevalence of low levels of physical activity and sedentary behaviour, and identify associated factors	*n* = 1619	37.7 (14.4, 18–86)	44.8%	12.4% borderline, 52.4% mild or moderate, 8.2% severe/profound, 27% unknown	25% DS, 12.2% autism, 12.7% cerebral palsy	Hours of TV watching (Proxy rater question): 3.42 (SD 2.13)	Age, sex, ethnicity, level of ID, ID-related conditions, general health status, days with activity limitation, chronic health conditions, obesity, depression, psychotropic medication use, epilepsy/seizure disorder, urinary incontinence, falls, mobility limitations, day/educational program or employment participation, residential type, social participation, special Olympics participation, low levels of PA	1.0
Melville et al. (2018) [27]	Scotland, adults with ID living in community	cross sectional, population- based sample	Assess the prevalence and correlates of screen time	*n* = 727	43.6 (NA, 18–90)	45%	mild 35.6%, moderate 26.5%, severe 17.9%, profound 20.0%	13.4% DS	Hours of screen time = watching TV, DVDs, videos or on the PC (C21st Health Check questionnaire- self and proxy report): 8.6% none, 2.8% 1–3 h/month, 14.3% <2 h/day, 23.3% 2–3 h/day, 28.1% 4–5 h/day, 22.8% 6 h/day	Sex, age, accommodation type, neighbourhood deprivation category, level of ID, Down syndrome, obesity, hearing impairment, visual impairment, mobility problems, mental ill health, problem behaviours, meets PA recommendation	1.0
Harris et al. (2018) [28]	Scotland, adults with ID living in the community	Secondary analysis of baseline data from two RCTs ^i^	Study correlates of objectively measured sedentary behaviour	*n* = 152	Mean age = N/A; 38.6% < 45 years, 61.4% ≥ 45 years	51.7%	48.3% mild, 35.7% moderate, 12.6% severe, 2.8% profound	N/A	Total sedentary time by ActiGraph GT3X+ accelerometer sedentary cut-off < 100 cpm): median 467.5 min/day (IQR ^j^ 411.0–542.2)	Age, sex, level of ID, physical health problems, mental health problems, problem behaviours obesity, accommodation type, neighbourhood deprivation	1.0

**^a^** SD standard deviation, **^b^** F females, **^c^** ID intellectual disabilities, ^d^ DS Down syndrome, ^e^ TV television, ^f^ WS Williams syndrome, ^g^ PWS Prader Willi syndrome, ^h^ BMI measured in kg/ m^2^, ^i^ RCT randomized controlled trial; ^j^ interquartile range, N/A not available from the details provided in the paper.

**Table 2:** Mapping the correlates of sedentary behaviour of adults with intellectual disabilities onto the ecological model.

Level	Category	Correlate	Association with SB a (Direction of Association)	No Association with SB
Individual	Physical, biological and genetic	Age		Oviedo et al., 2017 [25]; Hsieh et al., 2017 [26]; Harris et al., 2018 [28]; Melville et al., 2018 [27]
		Sex	Finlayson et al., 2011 [20] (women more sedentary); Hsieh et al., 2017 [26] (men more sedentary); Melville et al., 2018 [27] (men more sedentary)	Oviedo et al., 2017 [25]; Harris et al., 2018 [28]
		Ethnicity		Hsieh et al., 2017 [26]
		Genetic syndromes	Nordstrom et al., 2013 [24] (DS ^b^ −)	Hsieh et al., 2017 [26]; Melville et al., 2018 [27]
		Weight status	Nordstrom et al., 2013 [24] (−); Oviedo et al., 2017 (+) [25]; Hsieh et al., 2017 [26] (+); Melville et al., 2018 [27] (+)	Mikulovic et al., 2014 [23] Harris et al., 2018 [28] Hsieh et al., 2014 [21]
		Epilepsy	Hsieh et al., 2017 [26] (−); Melville et al., 2018 [27] (−)	
		Physical health	Harris et al., 2018 [28] (+)	Hsieh et al., 2017 [26]
		Psychotropic medication use		Hsieh et al., 2017 [26]
		Urinary incontinence		Hsieh et al., 2017 [26]
		Sleep habits	Mikulovic et al., 2014 [22] (+)	
		Mobility	Melville et al., 2018 [27] (+)	Hsieh et al., 2017 [26]
		Visual impairment		Melville et al., 2018 [27]
		Hearing impairment	Melville et al., 2018 [27] (−)	
		Falls		Hsieh et al., 2017 [26]
		Level of ID ^c^	Hsieh et al., 2017 [26] (−); Melville et al., 2018 [27] (−)	Harris et al., 2018 [28]
		Mental health	Harris et al., 2018 [28] (+)	Hsieh et al., 2017 [26]; Melville et al., 2018 [27]
		Problem behaviours		Harris et al., 2018 [28]; Melville et al., 2018 [27]
		Functional limitation in past 30 days		Hsieh et al., 2017 [26]
	Behavioural	Physical activity		Hsieh et al., 2017 [26]; Melville et al., 2018 [27]
		Special Olympics participation		Hsieh et al., 2017 [26]
	Socioeconomic status	Deprivation category		Harris et al., 2018 [28]; Melville et al., 2018 [27]
		Employment		Hsieh et al., 2017 [26]
Interpersonal		Living arrangements	Hsieh et al., 2017 [26] (family home +; foster home −)	Nordstrom et al., 2013 [24]; Harris et al., 2018 [28]; Melville et al., 2018 [27]
		Social participation		Hsieh et al., 2017 [26]
Environmental		Residential location (urban/rural)		Hsieh et al., 2017 [26]

^a^ SB sedentary behaviour, ^b^ DS Down syndrome, ^c^ ID intellectual disabilities.

## Supplementary Materials

The following are available online at http://www.mdpi.com/1660-4601/15/10/2274/s1, Table S1: PRISMA checklist, Table S2: Search strategy.
