# Supplementary material for: Correlates of Sedentary Behaviour in Adults with Intellectual Disabilities—A Systematic Review
Source: Int J Environ Res Public Health. 2018 Oct 17;15(10):2274. doi: 10.3390/ijerph15102274 (PMC6210806; doi:10.3390/ijerph15102274)
Supplement: Supplementary file 1 [file ijerph-15-02274-s001.pdf]

Supplementary files

Review

Correlates of Sedentary Behaviour in Adults with Intellectual Disabilities—A Systematic Review

Alyt Oppewal, Thessa I.M. Hilgenkamp, Liselotte Schäfer Elinder, Ellen Freiberger, Pauli Rintala, Myriam Guerra-Balic, Maria Giné-Garriga, Antonio Cuesta-Vargas, Guillermo R. Oviedo, Oriol Sansano-Nadal, Rocio Izquierdo-Gómez, Ingi Einarsson, Antti Teittinen, and Craig A. Melville

Table S1: PRISMA checklist.

Section/topic	#	Checklist item	Reported on page #
**TITLE** Definitions, measurement and correlates of sedentary behaviour in adults with intellectual disabilities – a systematic review			
Title	1	Identify the report as a systematic review, meta-analysis, or both.	1
**ABSTRACT**			
Structured summary	2	Provide a structured summary including, as applicable: background; objectives; data sources; study eligibility criteria, participants, and interventions; study appraisal and synthesis methods; results; limitations; conclusions and implications of key findings; systematic review registration number.	3
**INTRODUCTION**			
Rationale	3	Describe the rationale for the review in the context of what is already known.	4-6
Objectives	4	Provide an explicit statement of questions being addressed with reference to participants, interventions, comparisons, outcomes, and study design (PICOS).	6
**METHODS**			
Protocol and registration	5	Indicate if a review protocol exists, if and where it can be accessed (e.g., Web address), and, if available, provide registration information including registration number.	6

Eligibility criteria	6	Specify study characteristics (e.g., PICOS, length of follow-up) and report characteristics (e.g., years considered, language, publication status) used as criteria for eligibility, giving rationale.	6-7
Information sources	7	Describe all information sources (e.g., databases with dates of coverage, contact with study authors to identify additional studies) in the search and date last searched.	6
Search	8	Present full electronic search strategy for at least one database, including any limits used, such that it could be repeated.	29-30
Study selection	9	State the process for selecting studies (i.e., screening, eligibility, included in systematic review, and, if applicable, included in the meta-analysis).	7-8
Data collection process	10	Describe method of data extraction from reports (e.g., piloted forms, independently, in duplicate) and any processes for obtaining and confirming data from investigators.	8
Data items	11	List and define all variables for which data were sought (e.g., PICOS, funding sources) and any assumptions and simplifications made.	8
Risk of bias in individual studies	12	Describe methods used for assessing risk of bias of individual studies (including specification of whether this was done at the study or outcome level), and how this information is to be used in any data synthesis.	9
Summary measures	13	State the principal summary measures (e.g., risk ratio, difference in means).	N/A
Synthesis of results	14	Describe the methods of handling data and combining results of studies, if done, including measures of consistency	N/A
**Section/topic**	**#**	**Checklist item**	**Reported on page #**
Risk of bias across studies	15	Specify any assessment of risk of bias that may affect the cumulative evidence (e.g., publication bias, selective reporting within studies).	N/A
Additional analyses	16	Describe methods of additional analyses (e.g., sensitivity or subgroup analyses, meta-regression), if done, indicating which were pre-specified.	N/A
**RESULTS**			
Study selection	17	Give numbers of studies screened, assessed for eligibility, and included in the review, with reasons for exclusions at each stage, ideally with a flow diagram.	9-10, Figure 1
Study characteristics	18	For each study, present characteristics for which data were extracted (e.g., study size, PICOS, follow-up period) and provide the citations.	Tables 1 & 2
Risk of bias within studies	19	Present data on risk of bias of each study and, if available, any outcome level assessment (see item 12).	Table 1
Results of individual studies	20	For all outcomes considered (benefits or harms), present, for each study: (a) simple summary data for each intervention group (b) effect estimates and confidence intervals, ideally with a forest plot.	N/A

Synthesis of results	21	Present results of each meta-analysis done, including confidence intervals and measures of consistency.	N/A
Risk of bias across studies	22	Present results of any assessment of risk of bias across studies (see Item 15).	N/A
Additional analysis	23	Give results of additional analyses, if done (e.g., sensitivity or subgroup analyses, meta-regression [see Item 16]).	N/A
**DISCUSSION**			
Summary of evidence	24	Summarize the main findings including the strength of evidence for each main outcome; consider their relevance to key groups (e.g., healthcare providers, users, and policy makers).	18-22
Limitations	25	Discuss limitations at study and outcome level (e.g., risk of bias), and at review-level (e.g., incomplete retrieval of identified research, reporting bias).	22-23
Conclusions	26	Provide a general interpretation of the results in the context of other evidence, and implications for future research.	23
**FUNDING**			
Funding	27	Describe sources of funding for the systematic review and other support (e.g., supply of data); role of funders for the systematic review.	23

Table S2: Search strategy

Embase and MEDLINE - Ovid, 1990-

Search Terms		Citations Returned
1.	exp developmental disorder/ or intellectual impairment/ or exp mental deficiency/ or exp learning disorder/	
2.	exp developmental disabilities/ or exp intellectual disability/ or exp learning disorders/ or mentally disabled persons/	
3.	exp developmental disabilities/ or exp intellectual development disorder/ or "intellectual development disorder (attitudes toward)"/ or exp learning disabilities	
4.	((intellect$ adj3 (deficien$ or difficult$ or disab$ or disorder$ or impair$ or handicap$ or incapacit$ or handicap$ or sub?average or sub?norm$)) or (low$2 adj2 intellect$)).tw.	
5.	(learning adj3 (deficien$ or difficult$ or disab$ or disorder$ or handicap$ or impair$ or incapacit$ or handicap$ or sub?average or sub?norm$)).tw.	
6.	(mental$ adj3 (deficien$ or disab$ or handicap$ or impair$ or handicap$ or incapacit$ or retard$ or sub?average or sub?norm$)).tw.	
7.	((subaverage or sub$1 average or subnormal or sub$1 normal$) adj3 (cognit$ or intel$)).tw.	
8.	((development$ or neurodevelopment$) adj disab$).tw.	
9.	(education$ adj5 su?bnorm$).tw.	
10.	(cretin$ or feeble minded$ or imbecil$ or moron$).tw.	
11.	Or/ 1-10	
12.	Exp sedentary lifestyle/	
13.	sedentary behaviour.tw	
14.	sedentary behavior.tw	
15.	sedentary time.tw	
16.	Sedentariness.tw	
17.	Physical inactivity.tw	
18.	Exp television/	
19.	Exp video games/	

20.	"screen time".tw.	
21.	"sitting time".tw	
22.	"video games".tw.	
23.	“television watching”.tw	
24.	“television viewing”.tw	
25.	“video viewing”.tw	
26.	“electronic game playing”.tw	
27.	“computer gaming”.tw	
28.	“computer time”.tw	
29.	“computer use”.tw	
30.	“media time”.tw	
31.	“media use”.tw	
32.	“web browsing”.tw	
33.	“bedroom media”.tw	
34.	“electronic media”.tw	
35.	“PC”.tw	
36.	“PC use”.tw	
37.	“occupational sitting”.tw	
38.	“deskbound”.tw	
39.	“reading”.tw	
40.	“motor* transport”.tw	
41.	Or/ 12-40	
42.	11 and 41	

Web of Science

TS= screen time or television or computer games or video games or sitting time or sedentary behaviour or sedentary behaviour or sedentary time or sedentariness or sedentary lifestyle or physical inactivity

AND

TS= intellectual disab* or intellectual impair* or developmental disab* or learning disab* or mental retard* or mental handicap*

Google scholar

"intellectual disabilities" OR “intellectual disability” OR “developmental disability” or Developmental disabilities” OR “mental retardation” or “Mental handicap” AND "sedentary behaviour" OR "sedentary behaviour" OR "sedentary time" OR "sedentariness" OR "sedentary lifestyle" OR “physical inactivity”
